# Chronic Bronchitis and Current Smoking Are Associated with More Goblet Cells in Moderate to Severe COPD and Smokers without Airflow Obstruction

**DOI:** 10.1371/journal.pone.0116108

**Published:** 2015-02-03

**Authors:** Victor Kim, Michelle Oros, Heba Durra, Steven Kelsen, Mark Aksoy, William D. Cornwell, Thomas J. Rogers, Gerard J. Criner

**Affiliations:** 1 Division of Pulmonary and Critical Care Medicine, Temple University School of Medicine, Philadelphia, Pennsylvania, United States of America; 2 Department of Pathology, Temple University School of Medicine, Philadelphia, Pennsylvania, United States of America; 3 Center for Inflammation, Translational and Clinical Lung Research, Temple University School of Medicine, Philadelphia, Pennsylvania, United States of America

## Abstract

**Background:**

Goblet cell hyperplasia is a classic but variable pathologic finding in COPD. Current literature shows that smoking is a risk factor for chronic bronchitis but the relationship of these clinical features to the presence and magnitude of large airway goblet cell hyperplasia has not been well described. We hypothesized that current smokers and chronic bronchitics would have more goblet cells than nonsmokers or those without chronic bronchitis (CB), independent of airflow obstruction.

**Methods:**

We recruited 15 subjects with moderate to severe COPD, 12 healthy smokers, and 11 healthy nonsmokers. Six endobronchial mucosal biopsies per subject were obtained by bronchoscopy and stained with periodic acid Schiff-Alcian Blue. Goblet cell density (GCD) was quantified as goblet cell number per millimeter of basement membrane. Mucin volume density (MVD) was quantified as volume of mucin per unit area of basement membrane.

**Results:**

Healthy smokers had a greater GCD and MVD than nonsmokers and COPD subjects. COPD subjects had a greater GCD than nonsmokers. When current smokers (healthy smokers and COPD current smokers, n = 19) were compared with all nonsmokers (nonsmoking controls and COPD ex-smokers, n = 19), current smokers had a greater GCD and MVD. When those with CB (n = 12) were compared to those without CB (n = 26), the CB group had greater GCD. This finding was also seen in those with CB in the COPD group alone. In multivariate analysis, current smoking and CB were significant predictors of GCD using demographics, lung function, and smoking pack years as covariates. All other covariates were not significant predictors of GCD or MVD.

**Conclusions:**

Current smoking is associated with a more goblet cell hyperplasia and number, and CB is associated with more goblet cells, independent of the presence of airflow obstruction. This provides clinical and pathologic correlation for smokers with and without COPD.

## Background

Chronic Obstructive Pulmonary Disease (COPD) is characterized by persistent, progressive airflow limitation.[1] COPD encompasses a spectrum of clinical and pathological phenotypes, including emphysema on one end of the spectrum with chronic bronchitis (CB) on the other. CB has been associated with multiple adverse outcomes, including increased exacerbations, lung function decline, and mortality.[2–6] The pathologic correlate is goblet cell hyperplasia (GCH), which has been shown in several studies to be present in COPD.[7, 8] Mucus burden has prognostic significance; one study in lung volume reduction surgery patients found that small airway mucous metaplasia inversely correlated with changes in lung function after surgery,[9] whereas another study found that the degree of small airway mucus luminal occlusion correlated with mortality.[10]

However, there is a growing recognition of the disconnect between respiratory symptoms and magnitude of GCH. Few studies have addressed the correlation between the clinical phenotype and pathology, and the ones that do address it have not shown much of a relationship. Prior studies have examined subjects with mild airflow obstruction only,[7] and another examined those with CB only.[8] One study in advanced emphysema patients found no relationship between cough and sputum symptoms and degree of small airway mucus impaction,[11] and an established pathologic measure of mucous gland hyperplasia has little to no correlation with clinical symptoms.[12] We sought to quantify goblet cell density and mucin volume density in moderate to severe COPD subjects and in those with and without CB. It is also recognized that smoking is the greatest risk factor for CB. We hypothesized that those with CB would have greater goblet cell density and mucin volume density compared to those without CB. We also hypothesized that current smokers would have more goblet cell density and mucin volume density independent of the degree of airflow obstruction.

## Methods

### Patient Selection

We recruited subjects with moderate to severe COPD, current smokers without airflow obstruction (heretofore referred to as healthy smokers), and healthy nonsmokers. This study was conducted in accordance with the amended Declaration of Helsinki. Institutional Review Board approval was obtained from the Temple University Institutional Review Board, protocol number 20567, and all subjects signed written informed consent. Table 1 summarizes the inclusion and exclusion criteria. COPD subjects needed to have an FEV_1_ between 30–60% predicted. Healthy smokers needed to be currently smoking, have no airflow obstruction, and have a smoking history of greater than 10 pack years. Subjects with allergic rhinitis, acute or chronic sinusitis, upper respiratory tract infection, or COPD exacerbation within 6 weeks of the screening visit were excluded to negate the effect of acute or chronic infection on goblet cell hyperplasia. To eliminate the effects of steroids, subjects taking inhaled or oral steroids had them discontinued for 4 weeks prior to enrollment.

**Table 1:** Inclusion and Exclusion Criteria.

Inclusion Criteria
Age between 40 and 70 years
Diagnosis of COPD or at risk for COPD
Smoking History >10 pack years
FEV_1_ 30%–60% (GOLD II/III group), normal FEV_1_ (GOLD 0)
English speaking

Exclusion Criteria
Diagnosis of chronic sinusitis or allergic rhinitis
Presence of other lung disease (including asthma)
Pregnancy
Sinusitis or URI within the last 6 weeks
COPD Exacerbation within 6 weeks of screening visit
Presence of infiltrate or mass on CT scan
Anticoagulation or antiplatelet therapy within 6 half lives of bronchoscopy
Known allergy to lidocaine

Subjects also were asked the following questions: “Do you cough on most days for 3 consecutive months or more during a 12-month period?” and “Do you bring up phlegm on most days for 3 consecutive months or more during a 12-month period?” Subjects were classified as having CB if they answered yes to both questions for 2 consecutive years.

### Goblet cell density and mucin volume density quantification

**Fig 1:**
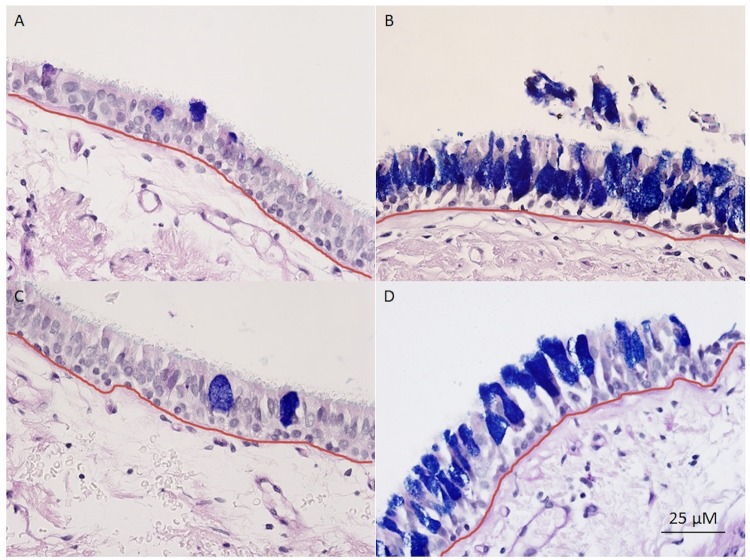
Examples of mucosal biopsies from A) a healthy nonsmoker, B) a healthy smoker, C) a COPD subject without chronic bronchitis, and D) a COPD subject with chronic bronchitis, taken at 40x. Specimens stained with periodic acid Schiff-Alcian Blue, staining goblet cells blue/purple. Basement membrane measured is outlined in red.

The subjects underwent bronchoscopy and 6 endobronchial mucosal biopsies were obtained, from the right lower, middle, and upper lobe bronchi. The specimens were embedded in paraffin and stained with periodic acid Schiff-Alcian blue. Goblet cells from all 6 specimens were counted and related to the length of basement membrane using Image J.[13] The resultant values were expressed as goblet cell density (cells/mm) as previously described.[8] Two investigators performed the measurements in a double blinded fashion (intraclass correlation coefficient of 0.744, p<.0001). Fig. 1 shows examples of a healthy nonsmoker and a COPD subject with CB. In order to assess goblet cell volume, mucin volume was measured using a modified model described by us ([14] using Image J. Length of basement membrane (L_BM_) and total area of mucin granules (MA) were measured. Mucin volume density (μL/mm^2^) was calculated using stereologic techniques as described previously:[15, 16] Mucin volume density = MA/(L_BM_)(4/π).

### Statistics

Statistics were performed using SPSS v21 (Cary, NC). Differences between groups (nonsmokers, healthy smokers, COPD) were assessed by one way ANOVA for continuous variables and Chi squared test for categorical variables. Individual groups were compared with Bonferroni test in a post hoc analysis. The correlation between goblet cell density and pack year history of smoking was assessed with Pearson’s correlation. Multivariate linear regression was performed with goblet cell density and mucin volume density as separate outcomes with current smoking, smoking pack year history, CB, demographic factors, and lung function as covariates. A p value of <0.05 was considered statistically significant.

## Results

The study population consisted of 38 subjects (11 nonsmokers, 12 healthy smokers, and 15 COPD subjects). Baseline characteristics are summarized in Table 2. The nonsmokers and healthy smokers were younger than COPD subjects. There were no differences in gender, race, or body mass index. Lung function, current smoking, and smoking history were by definition different between groups. There were no subjects with CB in the nonsmoker group, 2 (16.7%) in the healthy smoker group, and 10 (66.7%) in the COPD group. There were no differences in the presence of CB between active smokers and non- or ex-smokers.

**Table 2:** Baseline Characteristics.

	Nonsmokers	Healthy Smokers	COPD	
	n = 11	n = 12	n = 15	p
Age (years)	49.36±12.64	49.58±5.57	58.53±4.88	0.007
Gender (male, n(%))	6 (54.5)	4 (33.3)	12 (80)	0.053
BMI (kg/m^2^)	30.85±5.07	30.78±4.74	29.66±5.60	0.800
Race* (White, n (%))	5 (45.5)	2 (16.7)	2 (13.3)	0.136
FEV_1_ (% pred)	94.00±11.46	101.17±16.30	45.40±8.99	<0.0001
FVC (%pred)	93.00±15.28	103.67±16.87	77.73±16.26	0.001
FEV_1_/FVC	93.27±13.37	96.67±6.14	47.13±11.51	<0.0001
Smoking History (pack years)	0	25.58±10.66	29.00±14.77	<0.0001
Current Smoking (n, %)	0 (0)	12 (100)	7 (46.7)	<0.0001
Chronic Bronchitis (n, %)	0 (0)	2 (16.7)	10 (66.7)	0.001
Goblet Cells (n)	19.18±17.10	84.08±31.78	57.13±36.07	<0.0001
Length BM (mm)	7.04±2.87	9.47±3.52	10.02±3.89	0.101
Fields Measured (n)	7.09±2.81	8.83±3.01	8.00±3.48	0.426
GCD (cells/mm)	2.31±1.81	9.80±3.49	6.57±3.29	<0.0001
MVD (μL/mm^2^)	5.77±4.34	26.35±10.96	14.83±13.63	<0.0001

Definition of Abbreviations: BMI = body mass index, FEV_1_ = forced expiratory volume in 1 second, FVC = forced vital capacity, GCD = goblet cell density, MVD = mucin volume density.

*remainder of the cohort was African American.

**Fig 2:**
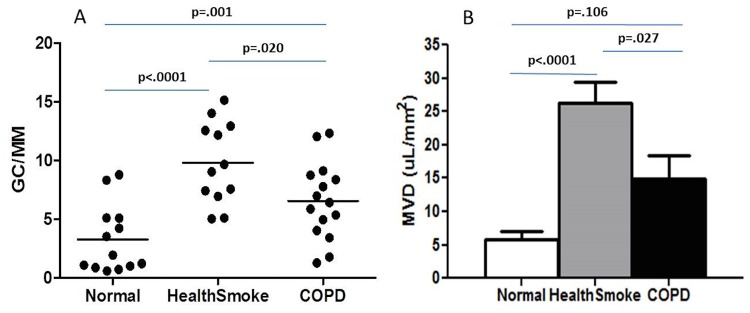
Goblet cell density in healthy nonsmokers, smokers without airflow obstruction, and COPD subjects. A. Data expressed as goblet cells per millimeter of basement membrane. Mucin volume density in the same three groups, B. Data expressed as mean±SE. Normal = healthy nonsmoking group, Healthsmoke = healthy smoker group, COPD = chronic obstructive pulmonary disease group, GC/MM = goblet cells per millimeter basement membrane, MVD = mucin volume density.

**Fig 3:**
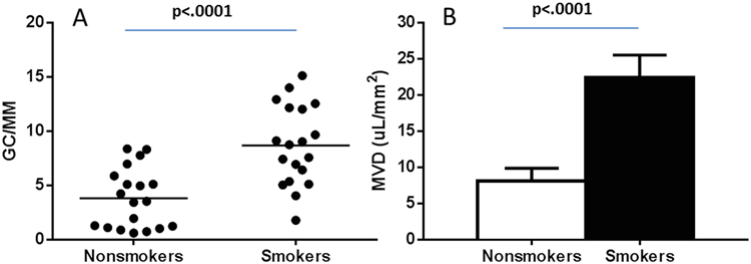
Goblet cell density in all smokers (smokers without airflow obstruction and COPD subjects that currently smoke) compared with all nonsmokers (healthy nonsmokers and COPD subjects who quit smoking). A. Data expressed as goblet cells per millimeter of basement membrane. Mucin volume density in all smokers compared with all nonsmokers, B. Data expressed as mean±SE. GC/MM = goblet cells per millimeter basement membrane, MVD = mucin volume density.

Healthy smokers (9.80±3.49 cells/mm) had a greater goblet cell density than nonsmokers (2.31±1.81 cells/mm, p<.0001). Healthy smokers (26.35±10.96 μL/mm^2^) also had a greater mucin volume density than nonsmokers (5.77±4.34 μL/mm^2^, p<.0001). Of considerable interest was that healthy smokers had a greater goblet cell density and mucin volume density compared to the COPD subjects (6.57±3.29 cells/mm, p = .020 and 14.83±13.63 μL/mm^2^, p = .027, respectively). See Fig. 2. When all smokers (healthy smokers and COPD current smokers combined, n = 19) were compared with all nonsmokers (nonsmokers and COPD ex-smokers, n = 19), the smokers had a greater goblet cell density (8.69±3.69 vs. 4.02±3.23 cells/mm, p<.0001) and mucin volume density (22.49±13.73 vs. 8.08±7.46 μL/mm^2^, p<.0001). All COPD ex-smokers quit smoking greater than 5 years prior to enrollment. See Fig. 3. In addition, there was a significant correlation between pack-year history of smoking with goblet cell density (r = .417, p = .010). The COPD group was analyzed separately, and there was no difference in GCD (7.19±3.97 vs. 6.74±3.21 gc/mm, p = 0.828) or MVD (12.57±11.54 vs. 16.71±16.10 μL/mm^2^, p = 0.629) between COPD smokers and COPD non-smokers.

**Fig 4:**
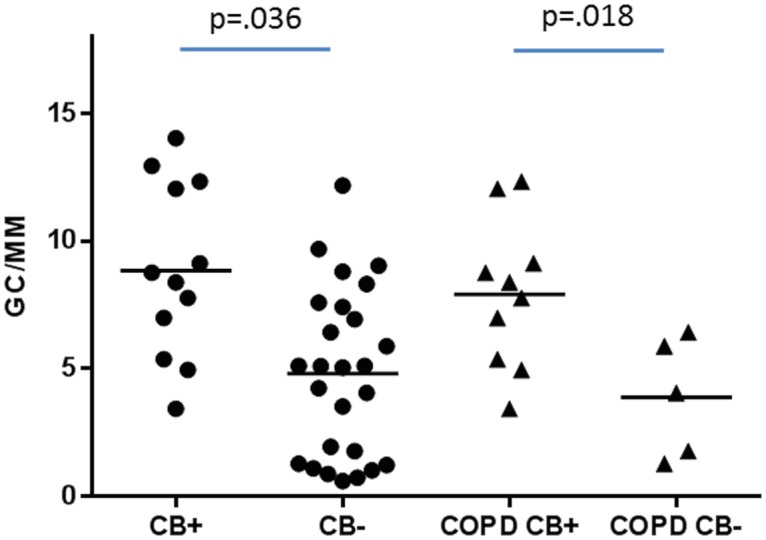
Goblet cell density between those with chronic bronchitis and those without chronic bronchitis in the entire cohort (left) and in the COPD subjects alone (right). Data expressed as goblet cells per millimeter of basement membrane. GC/MM = goblet cells per millimeter basement membrane, CB+ = chronic bronchitis, CB- = no chronic bronchitis, COPD CB+ = COPD subjects with chronic bronchitis, COPD CB- = COPD subjects without chronic bronchitis.

When those with CB (healthy smokers and COPD subjects included, n = 12) were compared to those without CB (in all 3 groups combined, n = 26), the CB group had greater goblet cell density (8.55±3.41 vs. 5.46±4.16 cells/mm, p = .036) but not mucin volume density (19.89±14.59 vs. 14.17±12.65 μL/mm^2^, p = .238). The COPD group was then analyzed separately. The differences between COPD CB+ and COPD CB- groups are summarized in Table 3. In comparison to the COPD CB- group (n = 5), the COPD CB+ group (n = 10) was older (57.77±5.52 vs. 58.00±3.46 years, p = .007), had a greater smoking history (28.88±9.92 vs. 17.60±3.91 pack years, p = 0.036), and had a lower FEV_1_ (43.33±7.65 vs. 54.40±8.05%predicted, p = .026). There were no significant differences in gender, racial distribution, or percentage of subjects currently smoking. The COPD CB+ group had a greater goblet cell density compared to the COPD CB- group (7.91±2.89 vs. 3.88±2.33 cells/mm, p = .018), but there was not a significant difference in mucin volume density (17.51±15.74 vs. 10.00±9.90 μL/mm^2^, p = .286). See Fig. 4.

**Table 3:** Characteristics of COPD patients with and without chronic bronchitis.

	COPD CB-	COPD CB+	
	n = 5	n = 10	P
Age (years)	58.00±3.46	57.77±5.52	0.007
Gender (male, n(%))	3 (60)	9 (75)	0.242
BMI (kg/m^2^)	30.81±5.81	29.08±5.71	0.242
Race* (White, n (%))	1 (20)	3 (30)	0.593
FEV_1_ (% pred)	54.50±8.05	43.33±7.65	0.026
FVC (%pred)	82.20±20.93	77.44±17.23	0.654
FEV_1_/FVC	53.20±8.58	45.55±12.14	0.240
Smoking History (pack years)	17.60±3.91	28.88±9.92	0.036
Current Smoking (n, %)	3 (60)	4 (40)	0.427
GCD (cells/mm)	3.86±2.33	7.91±2.89	0.018
MVD (μL/mm^2^)	10.00±9.90	17.51±15.74	0.286

Definition of Abbreviations: BMI = body mass index, FEV_1_ = forced expiratory volume in 1 second, FVC = forced vital capacity, GCD = goblet cell density, MVD = mucin volume density.

*remainder of the cohort was African American.

In multivariate linear regression, both CB and current smoking were significant determinants of goblet cell density (standardized beta coefficients. 419, p = .019 and. 466, p = .002, respectively). The variables pack year history of smoking, age, gender, race, and lung function did not reach statistical significance. For mucin volume density, the only statistically significant covariate was current smoking (standardized beta coefficient. 428, p = .018). See Table 4.

**Table 4:** Multivariate Linear regression for Goblet Cell Density and Mucin Volume Density.

	GCD			MVD		
	Standardized Beta Coeff.	t	p	Standardized Beta Coeff.	t	p
Current Smoking (yes vs. no)	0.466	3.464	0.002	0.428	2.528	0.018
Chronic Bronchitis (yes vs. no)	0.419	2.501	0.019	0.256	1.233	0.228
Smoking History (pack-years)	0.186	1.163	0.255	0.094	0.487	0.630
Age (per year)	-0.057	-0.367	0.717	-0.140	-0.890	0.381
FEV1 (% predicted)	0.148	1.037	0.309	-0.019	-0.074	0.942
FVC (% predicted)	0.156	1.154	0.258	0.204	0.835	0.411
Sex	0.190	0.647	0.523	0.176	1.099	0.282
Race	-0.089	-0.386	0.703	0.035	0.215	0.831

## Discussion

We used two different measures of goblet cell hyperplasia (goblet cell density for goblet cell number and mucin volume density to assess for hyperplasia) to show that smokers without airflow obstruction and COPD subjects had a greater goblet cell density compared to nonsmokers, and that smokers without airflow obstruction had a greater goblet cell density and mucin volume density compared to COPD subjects. We also showed that active smokers and chronic bronchitics had a greater goblet cell density compared to nonsmokers and those without chronic bronchitis, respectively. This difference was observed regardless of the presence or absence of airflow obstruction, overall smoking burden, or demographic factors. This study is unique by virtue of the clinical correlation to large airway pathology with segregation of subjects by chronic bronchitis and current smoking.

Prior studies have established that GCH is greater in COPD subjects compared to those without airflow obstruction. Innes et al. performed a similar study of smokers with and without airflow obstruction and found significantly increased goblet cell density compared to nonsmokers, and those with mild airflow obstruction had more goblet cells compared to the smokers without airflow obstruction.[7] Saetta et al. found an increased number of goblet cells in the peripheral airways of COPD subjects with CB compared to those without airflow obstruction.[8] In addition, the expression of MUC5AC and MUC5B, two predominant airway mucins, were increased in COPD subjects as compared to controls.[17] We have shown that mucin volume density increased incrementally as COPD disease severity increased.[14] A large pathologic study revealed that the number of small airways occluded by mucus increased with greater degrees of airflow obstruction.[18] However, literature on the differences between nonsmokers and current smokers and on the differences between those with CB and those without is sparse.

Our study differs from prior bronchoscopic studies by the inclusion of COPD subjects (current and ex-smokers) with moderate to severe airflow obstruction and those with and without CB. Other studies of large airway goblet cell hyperplasia in COPD included subjects with mild to moderate disease.[8] We chose subjects with moderate to severe disease because this group is more prone to COPD exacerbations, their goblet cell pathology has been poorly described in other bronchoscopic studies, and they can safely undergo bronchoscopy.[19] Although we showed that the COPD subjects had a greater goblet cell density compared to nonsmokers, we also showed that smokers without airflow obstruction, in comparison to the COPD subjects and normal controls, had the highest goblet cell density and mucin volume density, which differs from prior studies. Based on these results and previously reported data,(7) our data suggests that large airway epithelial mucin stores increase initially before the onset of mild airflow obstruction and then decrease as more severe disease develops. In our cohort, this observation is predominantly driven by the presence of active smoking; active smokers had an increased goblet cell density and mucin volume density, and a weak but significant linear relationship existed between goblet cell density and smoking history.

What also sets this study apart from prior ones is the demonstration of greater goblet cell density in chronic bronchitics versus those without CB, in the COPD group but also in the smokers without airflow obstruction. Thurlbeck et al. demonstrated that peripheral airway mucous gland hyperplasia and airway mucus were increased in CB but failed to show an increase in goblet cell metaplasia in chronic bronchitics with little airflow obstruction.[12, 20] The study by Saetta et al. included subjects with CB alone, making the detection of pathologic differences between those with and without CB impossible. [8] Other studies failed to show any differences in the Reid Index, an established measure of submucosal gland hypertrophy, between those with CB and those without.[12, 21]

Smoking has been associated with CB in many prior studies.[3, 5, 22, 23] We have shown that in subjects with moderate to severe COPD, those with CB were more likely to be current smokers.[3] A thirty-year observational study of 1,711 Finnish men found that smoking resulted in a cumulative incidence of CB of 42%.[5] A meta-analysis pooling numerous studies showed that current smoking conferred a relative risk of 3.41 for the development of chronic bronchitis.[22] However, few studies have addressed differences in goblet cell pathology between smokers and nonsmokers. Whereas some studies have shown increases in goblet cell hyperplasia in smokers compared with nonsmokers,[7, 21] others have not.[8] In this study, we demonstrated that current smokers, independent of airflow obstruction, had increased goblet cell density and mucin volume density compared to nonsmokers or ex-smokers, a rather novel finding. This finding remained significant on multivariate analysis for both measures of goblet cell hyperplasia. Indeed, lung function, as measured by FEV_1_ and FVC, did not have significant bearing on GCD or MVD in multivariate analysis. This suggests that goblet cell hyperplasia does not contribute to airflow obstruction but rather that active smoking is its primary determinant.

Similarly, goblet cell hyperplasia has been shown in CB,[7, 8, 24] but few studies have compared goblet cell density in COPD subjects with CB compared to those without it. Many studies about COPD pathology have either studied COPD undifferentiated by phenotype[14, 18] or chronic bronchitics alone.[8, 24] To make matters worse, the existing literature showing the link between symptoms of cough and phlegm and large airway pathology is weak at best.[25] This study improves our current understanding of the clinico-pathologic link between CB and airway pathology.

We feel that the cohort used in this study adds strength to our findings. We purposefully excluded individuals with conditions linked or thought to be associated with lower airway goblet cell hyperplasia (upper airway disease, recent exacerbations, etc.). In addition, we did not include subjects actively treated with inhaled steroids to eliminate the confounding nature of this treatment on goblet cells. Finally, bronchoscopic literature on those with moderate to severe disease is rare, and these results offer a greater contribution our current understanding.

While this study has its strengths, there are also some limitations. One can argue that the study subgroup populations are small, particularly when dividing the COPD subjects into CB+ vs. CB-. By the nature of the study, we only sampled large airways, and therefore could not comment on small airway disease. We did not include subjects with mild to moderate airflow obstruction, as in prior literature. We excluded subjects on inhaled or oral steroids whom we considered unsafe to have them discontinued, thereby removing frequent exacerbators from the study. In addition, exacerbation history was not collected prior to enrollment in a systematic fashion. Finally, while both measures of goblet cell hyperplasia were significantly different between current smokers and non- or ex-smokers, only one was found to be different between those with and without CB. These observed differences are up for speculation, but suggest that goblet cell number and volume are greater as a result of active smoking, where goblet cell number alone is greater in chronic bronchitics. The reasons for these differences are unknown. Finally, it is unclear if the goblet cell density causes the clinical phenotype of CB or if they are purely associated.

## Conclusions

Our study offers insight into the correlation between airway pathology and clinical phenotype. We were able to demonstrate in this small sample that current smoking and CB, and not airflow obstruction, were independent factors associated with goblet cell hyperplasia. These findings have significant clinical implications, underscoring the importance of smoking cessation and identifying a phenotype at higher risk for poor outcomes. However, more subjects need to be analyzed in order to make a more conclusive determination of the links between airflow obstruction and smoking on goblet cell hyperplasia.
